# Effect of non-stoichiometry of initial reagents on morphological and structural properties of perovskites CH_3_NH_3_PbI_3_

**DOI:** 10.1186/s11671-018-2841-6

**Published:** 2019-01-05

**Authors:** Anatolii Belous, Sofiia Kobylianska, Oleg V’yunov, Pavlo Torchyniuk, Volodymyr Yukhymchuk, Oleksandr Hreshchuk

**Affiliations:** 1 V.I. Vernadskii Institute of General and Inorganic Chemistry of the NAS of Ukraine, Kyiv, Ukraine; 2 V. Lashkaryov Institute of Semiconductor Physics, NAS of Ukraine, Kyiv, Ukraine

**Keywords:** Metal halide perovskite, Film, Microstructure, Chemical reaction, Raman spectroscopy, 81.07.Pr, 81.07.–b, 81.70.Jb, 87.64.kp

## Abstract

The properties of films of organic-inorganic perovskites CH_3_NH_3_PbI_2.98_Cl_0.02_ depending on the ratio of starting reagents in solutions (PbI_2_:{CH_3_NH_3_I + CH_3_NH_3_Cl}) has been investigated. It was found that the formation of the perovskite structure with the ratio of the initial reagents PbI_2_: CH_3_NH_3_I = 1:1 occurs at 70–80 °C, and with the increase of the temperature of thermal treatment to 120 °C, the thermal destruction of the perovskite begins. When the ratio of the starting reagents PbI_2_: CH_3_NH_3_I = 1:2, the formation of the perovskite structure occurs through the intermediate compound (CH_3_NH_3_)_2_PbI_4_, and when the ratio is 1:3—(CH_3_NH_3_)_3_PbI_5_ and (CH_3_NH_3_)_2_PbI_4_. Independent on the ratio of the initial components (CH_3_NH_3_I:PbI_2_), the ratio between the content of lead and iodine in the films remains unchanged, that is why a significant difference in the film properties could be explained by the anisotropy of the particle shape, which is consistent with the data of electron microscopy and X-ray diffractometry.

**Electronic supplementary material:**

The online version of this article (10.1186/s11671-018-2841-6) contains supplementary material, which is available to authorized users.

## Background

Nowadays, solar energy is emerging as alternate sources of energy and the development of technologies to transform renewable energy into electricity is essential to societal advancement [1]. The most widely commercialized solar cells based on crystalline or multicrystalline silicon and semiconductor CuIn, GaSe_2 − x_S_x_, CdTe [2]. In practice, the most solar cells are based on silicon (85–90%) [3]. The theoretical power conversion efficiencies (PCE) of these solar modules are as high as 28–19.9%. However, for commercialized solar modules, PCE is only 18% for crystalline silicon solar cells and 12–14% for polycrystalline Si. The main disadvantage of silicon and semiconductor’s based solar cells is the narrow spectral range of sensitivity to solar radiation and their indirect bandgap [4]. This causes the use of a large thickness (~ 100 μm) of the active layer to increase the amount of absorption of solar radiation and, consequently, leads to a relatively high cost.

Promising new class of solar cell is the perovskite one, which has drawn the considerable interest of the researchers due to a remarkable rapid growth of its PCE. Organic-inorganic perovskites (OIP) are a class of substances with typical chemical formula ABX_3_, where A is an organic cation (often methylammonium CH_3_NH_3_^+^, formamide CH(NH_2_)_2_^+^), B is an inorganic cation (usually Pb^2+^), and X is a halide anion (I^−^, Cl^−^or Br^−^) [5, 6]. Synthesis of these compounds is relatively easy, and they have high photoelectric characteristics, in particular, the large diffusion length of charge carriers [7]. An impressive increase in PCE for solar cells based on OIP from ~ 3.4% in 2004 to 23.3% (22.6% certified) in early 2018 [8–10] has generated a considerable interest in the study of its properties. Significant achievements were obtained due to the development of novel technology for the formation of these compounds, which allow production of smooth and dense active layers of high-performance photovoltaic devices [11, 12]. The process of forming a smooth film without pores requires careful control of the chemistry of solutions of precursors and the conditions for their deposition [13–15]. In particular, deposition of a stoichiometric amount of methylammonium and lead iodides (MAI:PbI_2_ = 1:1) on a glass substrate does not allow preparation of a dense film of methylammonium lead iodide perovskites (MAPbI_3_), since in this case, the needle-like crystals grow. This film morphology significantly reduces the PCE. At the same time, using additional (super-stoichiometric) amount of MAI, a dense film can be prepared [16, 17].

Several fundamental properties make OIP extremely promising for photovoltaic applications, including low defect density, long charge carrier lifetime and diffusion length, low speed of recombination, and high optical absorption coefficient due to direct band gap [18, 19]. However, to date, many of the fundamental properties of OIP have not yet been studied in detail. It is known that one of the main drawbacks of this class of materials is their low stability. Exposure to even ambient atmospheric conditions causes severe degradation of OIP, and their unique optoelectronic properties diminish consequently. Numerous works have established the effects of moisture and oxygen, heat treatment at *T* > 100 °С, and the action of UV radiation ingress into MAPbI_3_ films, and it is well understood that as MAI is evaporated, solid PbI_2_ remains on the film [20–26]. This instability not only complicates the successful implementation of solar cells based on MAPbI_3_, but also the study of the properties of this material. In particular, the X-ray diffraction analysis, Raman and photoluminescence (PL) studies with a large exposure time (> 6 s) leads to the destruction of the perovskite. Therefore, it is important to take into account these features in the study of OIP and to distinguish spectra of the materials and products of their decomposition under abovementioned factors. It should be noted that despite the degradation of this material under the influence of external factors, the number of works devoted to the study of its properties significantly increases each year [27–29]. This may indicate that the scientific community believes in the possibility of using perovskites in solar cells.

As noted above, the perovskite MAPbI_3_ was extensively investigated by various methods, but today, there is little data on the influence of non-stoichiometric quantities of reagents on the properties of synthesized crystals. At the same time, the presence of various complexes (PbI^3−^, PbI_4_^2−^) in the solution used for the synthesis of organic-inorganic perovskites affects the microstructure of the resulting film [30, 31]. In particular, the change in the ratio of CH_3_NH_3_I:PbI_2_ from 1:1 to 1:3 in the initial solutions leads to significant changes in the microstructure and properties of the films [16, 32]. The investigation of the parameters of devices based on organic-inorganic perovskites CH_3_NH_3_PbI_3-x_Cl_x_ showed that with increasing MAI excess in the initial solution in the range from 1 to 3, values of open circuit voltage (Voc) increase, and the short circuit current density (Jsc), fill factor (FF), and power conversion efficiency (PCE) pass through a maximum at MAI excess of ~ 2–2.6 [33]. Therefore, the study of chemical and physical mechanisms, which, with non-stoichiometry of the starting reagents, significantly affect their morphological and structural properties, is very important both from the fundamental point of view and for the practical application of perovskites.

To study the influence of non-stoichiometry of the starting reagents on the properties of synthesized OIP, the Raman spectroscopy and X-ray diffractometry have been used. Raman spectroscopy is a sensitive and rapid method for diagnosing various compounds both in the form of solutions and in solids. Investigation of OIP by Raman spectroscopy and X-ray diffractometry methods can significantly expand the existing understanding of the processes of their formation, the features of the crystalline structure, and its effect on the film morphology.

In this paper, we aim to study the formation of films of organic-inorganic perovskite CH_3_NH_3_PbI_2.98_Cl_0.02_ and the influence of different ratios of the starting reagents (CH_3_NH_3_I: PbI_2_) on their microstructure.

## Methods

### Methods of synthesis

Lead iodide (PbI_2_), methylammonium chloride CH_3_NH_3_Cl, and pre-synthesized methylammonium iodide CH_3_NH_3_I were used as starting materials. To stabilize the perovskite structure, the partial substitution of iodine with chlorine was carried out by the addition of methylammonium chloride CH_3_NH_3_Cl [16, 34]. Dried dimethylformamide (DMF) was used as the solvent.

For the deposition of CH_3_NH_3_PbI_2.98_Cl_0.02_ films (MAPbI_3-x_Cl_x_), the starting reagents PbI_2_, CH_3_NH_3_I, and CH_3_NH_3_Cl in ratios of 1:0.98:0.02 (hereinafter 1:1); 1:1.98:0.02 (1:2); 1:2.98:0.02 (1:3) was dissolved in DMF and stirred at 70 °C for 1 h. The films were deposited in a dry box. The previously obtained clear solution was deposited to the purified glass substrate and to the FTO (fluorine-doped tin oxide) substrates by spin-coating at 1200 rpm for 30 s. Thermal treatment of films was carried out on a preheated hot plate at temperatures from 70 to 180 °C for 30 min.

### Characterization

The microstructure of starting reagents (PbI_2_ і CH_3_NH_3_I) and OIP (CH_3_NH_3_PbI_3_) was studied using a scanning electron microscope SEC miniSEM SNE 4500 MB. The elemental composition of the films was determined using an EDAX Element PV6500/00 F spectrometer, which is included in the set of this microscope.

The phase composition of films was identified by X-ray powder diffractometry (XRPD) using a DRON-4-07 diffractometer (CuKα-radiation, 40 kW, 18 mА) over 2Θ = 10–120°, a step of 0.04°, and a count time of 4 s. Structural parameters were determined by the Rietveld profile analysis method using XRPD data. Raman spectra were excited by 532 and 671 nm lines of solid state lasers and acquire usage of single stage monochromator equipped with charge-coupled device (CCD) detector (Andor). The exciting laser power was kept as low as possible, to avoid the damage of molecules under investigation either due to heating or photochemical reactions.

## Results and discussion

### Investigation of solutions

**Fig. 1:**
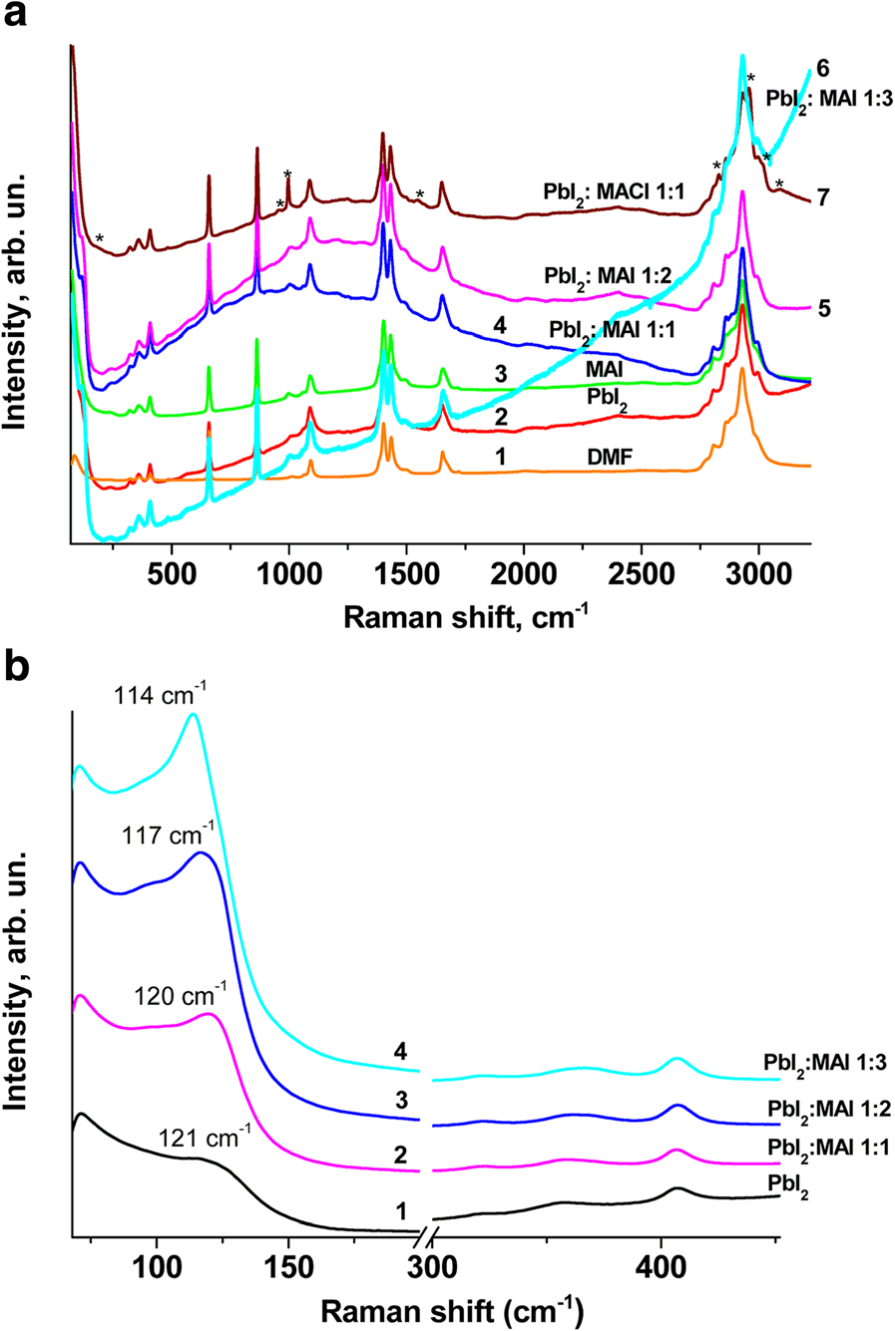
**a** Raman spectra of a solvent DMF (curve 1) and dissolved in it compounds: 2 - PbI_2_, 3 - CH_3_NH_3_I, 4 - PbI_2_ and CH_3_NH_3_I (1:1), 5 - PbI_2_ and CH_3_NH_3_I (1:2), 6 - PbI_2_ and CH_3_NH_3_I (1:3), 7 - PbI_2_ and CH_3_NH_3_Cl (1:1). **b** Raman spectra of solutions: 1- PbI_2_, 2 - PbI_2_ and CH_3_NH_3_I (1:1), 3 - PbI_2_ and CH_3_NH_3_I (1:2), 4 - PbI_2_ and CH_3_NH_3_I (1:3) in DMF in the low-frequency range. All spectra were obtained with *λ*_exc_ = 532 nm at room temperature

Figure 1 a, b show Raman spectra of pure DMF (curve 1) and dissolved compounds such as PbI_2_ (curve 2), CH_3_NH_3_I (curve 3), PbI_2_ and CH_3_NH_3_I in the ratio 1:1 (curve 4), PbI_2_ and CH_3_NH_3_I in the ratio 1:2 (curve 5), PbI_2_ and CH_3_NH_3_I in the ratio 1:3 (curve 6), and PbI_2_ and CH_3_NH_3_Cl in the ratio 1:1 (curve 7) obtained by laser excitation at *λ* = 532 nm at room temperature. It should be noted that the solutions of both PbI_2_ and CH_3_NH_3_I in DMF are practically transparent. At the simultaneous dissolution of PbI_2_ and CH_3_NH_3_I in DMF the coloring of a solution changes from light yellow at a ratio of components (1:1) to a dark yellow (1:3). The coloring of the solution shows that there is a chemical interaction between the components of PbI_2_ and CH_3_NH_3_I.

Sufficiently intense bands appear in the spectral region from 50 to 3500 cm^−1^ in the Raman spectrum of DMF (curve 1). Almost all the same bands occur in the spectrum of the solution of PbI_2_ in DMF (curve 2), except one, which is a manifestation of the vibration mode of Pb-I with a frequency of ~ 114 cm^−1^ and some features in the region of 475 cm^−1^. Only the Raman bands of DMF (curve 3) appear in the spectrum of the solution of CH_3_NH_3_I in DMF.

In the spectra of solutions in which both PbI_2_ and CH_3_NH_3_I compounds were added in the ratio 1:1 and 1:2, except for bands with frequencies of 114 cm^−1^, broad bands appear with maxima at 1000 and 1250 cm^−1^ (Fig. 1a, curves 4, 5), respectively. For the spectrum of the solution in which the PbI_2_ and CH_3_NH_3_I compounds were added in the ratio of 1:3, the maximum shifts to a long-wave region (Fig. 1a, curve 6). It is most likely that all of them appear due to the contribution of photoluminescence from the formed compound CH_3_NH_3_PbI_3_ because when the Raman spectra are excited with radiation *λ* = 671 nm, they do not appear in the spectra (spectra are shown in Additional file 1).

As noted above, characteristic Pb-I vibration band appears in the range 114–121 cm^−1^ (Fig. 1b) in all Raman spectra of solutions with different ratios of PbI_2_ and CH_3_NH_3_I compounds. Its relative intensity increases and the maximum of the bands are shifted to the low-frequency side with an increase in CH_3_NH_3_I content in the solution (Fig. 1b). This shift of the Raman peak correlates with the shift of the optical absorption edge from 2.54 eV for PbI_2_ in DMF down to 2.24 eV for the mixture of PbI_2_ and CH_3_NH_3_I mixed in the ratio of 1:3 (spectra are added to the Additional file 1: Figures S1 and S2). These spectral changes indicate that adding of CH_3_NH_3_I increases the probability of forming lead polyiodides, such as [PbI_3_]^−1^, [PbI_4_]^−2^, [PbI_5_]^−3^, and [PbI_6_]^−4^. Our results correlate with the result of work [31], where the influence of precursors on the structural and optical properties of the perovskites was shown. The different composition of polyiodides can cause the different morphology of the perovskite films, including those observed in our work. Since a small fraction of CH_3_NH_3_Cl (2% relative to CH_3_NH_3_I) was added to the solution together with CH_3_NH_3_I, it was necessary to establish the possible contribution of this compound to the Raman spectra. For this purpose, the Raman spectrum of CH_3_NH_3_Cl in DMF (Fig. 1a, curve 7) was registered. It shows a series of additional bands with the following frequencies: 178, 953, 997, 1547, 2829, 2957, 3020, and 3092 cm^−1^, which in Fig. 1a, curve 7 are marked with asterisks. Indeed, these bands’ frequencies are close to the frequencies of the Raman bands of the compound CH_3_NH_3_PbICl_2_, obtained in [35]. However, the abovementioned bands are not manifested in the Raman spectra of CH_3_NH_3_PbI_2.98_Cl_0.02_ solutions due to a small fraction of chlorine atoms.

### Investigation of films

**Fig. 2:**
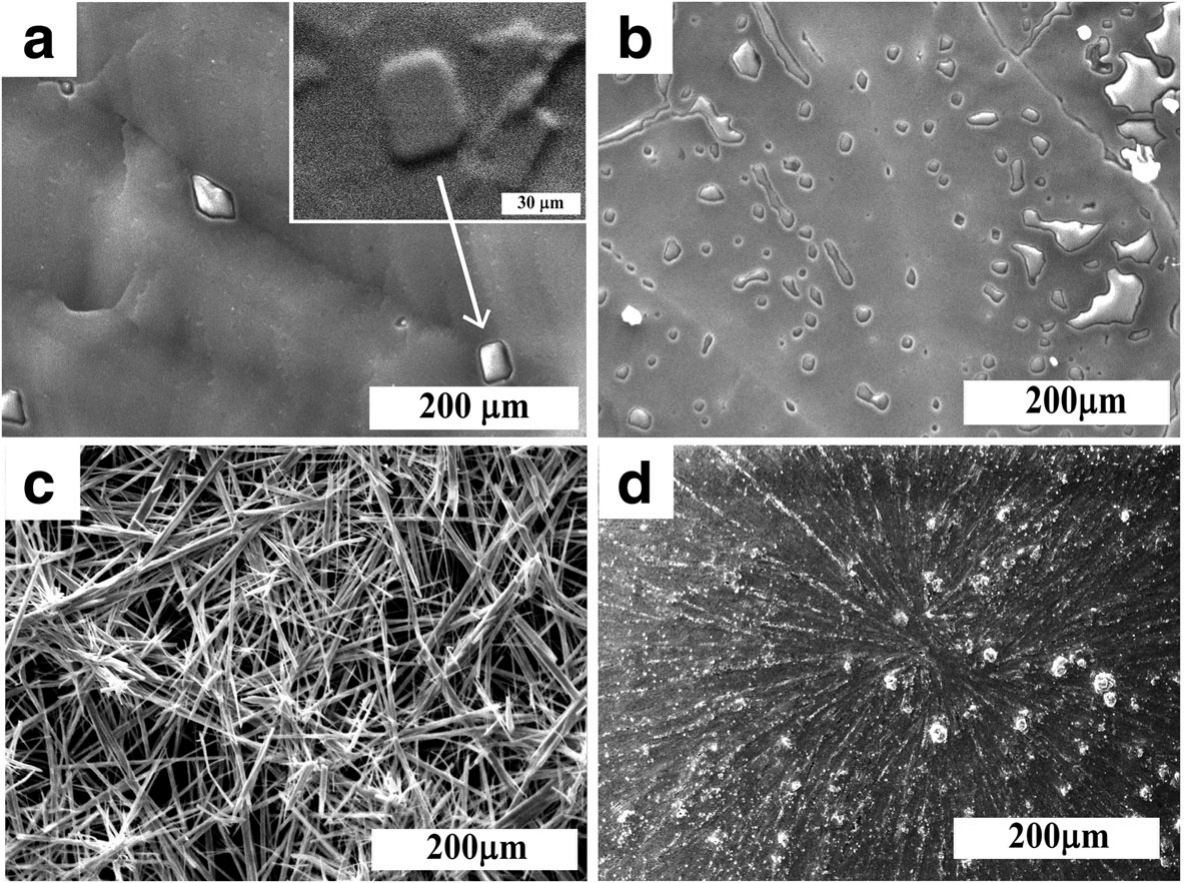
Images of CH_3_NH_3_I (**a**, **b**) and PbI_2_ (**c**, **d**) films without heating (**a**, **c**) and after thermal treatment at 90 °С (**b**, **d**), deposited on glass substrates. Inset in **a**—increased SEM-BSE image of heterogeneity

Figure 2 shows images of the films of initial reagents deposited on the glass substrates surface.

The microstructure of CH_3_NH_3_I looks like glass with small heterogeneities of a certain shape on the surface (Fig. 2a). Microscopic and energy-dispersive X-ray spectroscopy (EDX) studies have shown that films are thinner in the area of heterogeneities BSE (backscattering electrons) analysis suggests that this is due to increasing the surface level in these places (see EDX spectra in Additional file 1). Such areas are probably formed due to the rapid evaporation of the solvent from the film. The significant increase in the number of such heterogeneities with temperature increase to 90 °C confirms this fact (Fig. 2b).

When PbI_2_ solution is deposited at room temperature (without heating), large elongated (wire-like [36]) grains grow in all directions (Fig. 2c). At 90 °C, initially, the wire-like grains grow from a small number of crystallization centers. Further, the supersaturated solution is formed, and grains grow in supersaturated regime [37, 38] with the initially formed wire-like grains as seed particles (Fig. 2d).

**Fig. 3:**
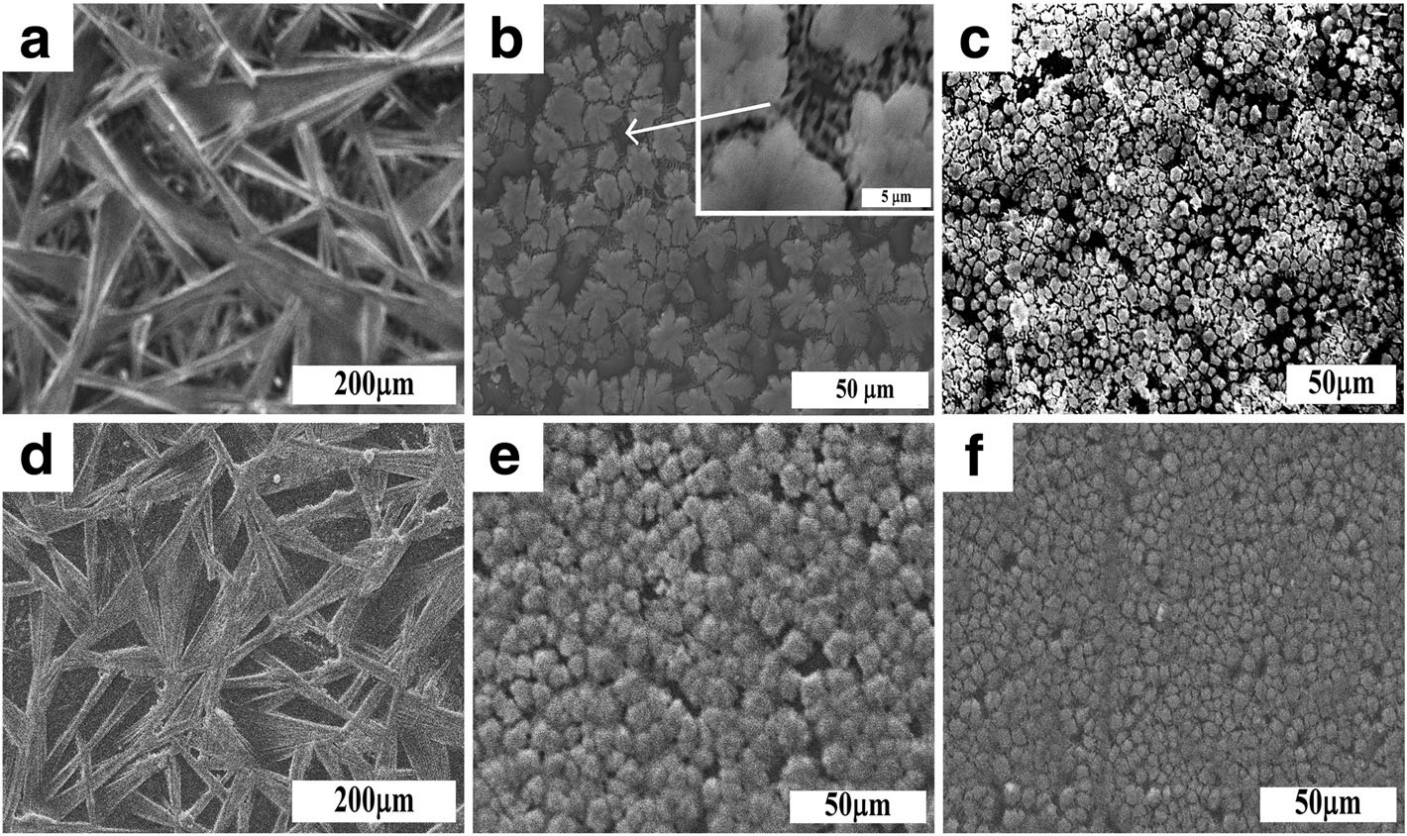
Images of organic-inorganic perovskites films deposited on glass substrates (**a**-**c**) and FTO/glass (**d**-**f**). Inset in **b**—the enlarged image of the intergrain area

Figure 3 shows the surface of the films of organic-inorganic perovskites that were deposited on the glass substrate and FTO/glass. When initial reagents (CH_3_NH_3_I:PbI_2_) were taken in the ratio 1:1, the microstructure of the organic-inorganic perovskite film deposited on the glass substrate and FTO/glass practically does not differ: there are structured films with a significant anisotropy of the particle shape (needle-like). In the case of a ratio of initial reagents 1:2, particles in the form of a maple leaf are visible on the glass. The growth of the latter occurs from the center of crystallization in 5–6 directions. Between large particles, small leaf-like particles appear (see insert on Fig. 3b). At the same time, after deposition of the film on FTO/glass surface, particles become more isotropic in the form. This agreed with the data of Ref. [39], where a strong difference in microstructures is observed for films deposited on different polycrystalline and amorphous substrates. In the case of the ratio of initial reagents 1:3, the size of the particles is significantly reduced and a more dense film is formed.

**Fig. 4:**
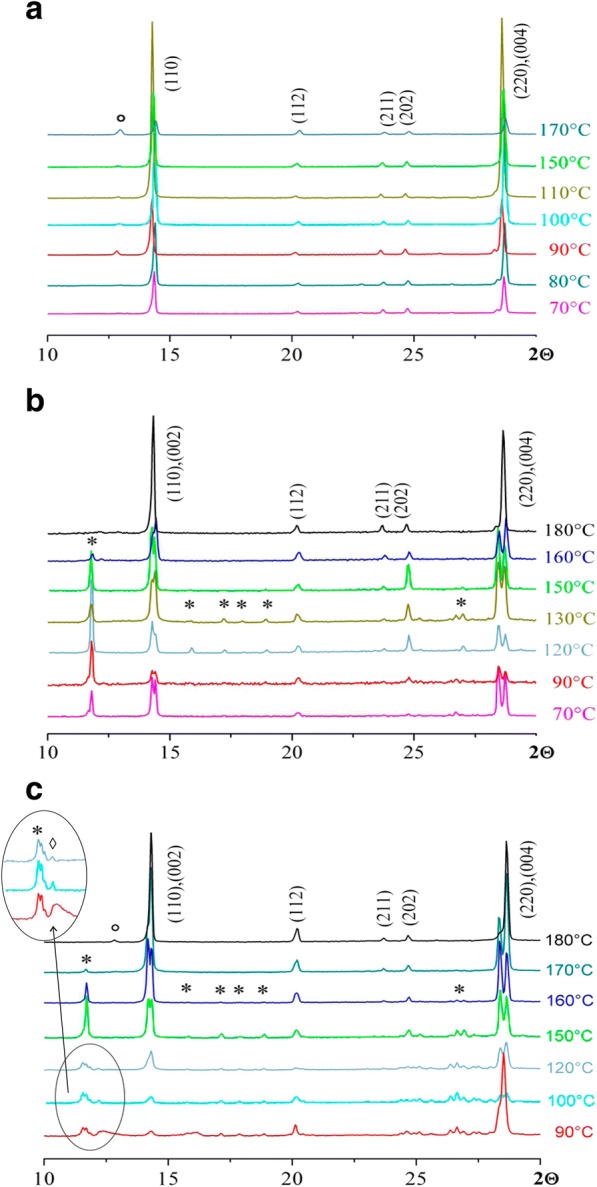
X-ray diffraction pattern of CH_3_NH_3_PbI_3_ films obtained with different ratios of the starting reagents PbI_2_ and CH_3_NH_3_I: **a** 1:1. **b** 1:2. **c** 1:3.Asterisk denotes (CH_3_NH_3_)_2_PbI_2_; diamond denotes (CH_3_NH_3_)_3_PbI_5_

Figure 4 shows the results of the XRPD analysis of films after heat treatment in the temperature range from 70 to 180 °C.

For the system with the ratio of the initial reagents PbI_2_:CH_3_NH_3_I = 1:1, it was found that the single-phase product is formed at 70–80 °С by the reaction:

1 $$ \mathrm{PbIz}+\mathrm{CH}3\mathrm{NH}3\mathrm{I}\overset{70-80{}^{\circ}\mathrm{C}}{\to}\mathrm{C}\mathrm{H}3\mathrm{NH}3\mathrm{PbI}3. $$

The synthesis of CH_3_NH_3_PbI_3_ films was carried out in a glove box, that is why the formation of phases of mono- and dihydrates (CH_3_NH_3_PbI_3_·H_2_O, (CH_3_NH_3_)_4_PbI_6_·2H_2_O), which are typical for the synthesis in a humid atmosphere, was not observed (Fig. 4a) [40, 41].

Increasing of the temperatures leads to the appearance of PbI_2_ peaks (2Θ = 12.8 °), which can be explained by the partial decomposition of the perovskite. It has been shown that other possible products of the decomposition of perovskite CH_3_NH_3_PbI_3_, except for the phase of PbI_2_, are CH_3_NH_2_ and HI [42, 43]. Authors [44] have been shown that in the Fourier-transform infrared spectroscopy (FTIR) spectra of the products, there are bands indicating the presence of a C-I bond. Therefore, the reaction of the decomposition of organic-inorganic perovskite can be written as:

2 $$ \mathrm{CH}3\mathrm{NH}3\mathrm{PbI}3\overset{>80{}^{\circ}\mathrm{C}}{\to}\mathrm{PbI}2+\mathrm{CH}3\mathrm{I}\uparrow +\mathrm{NH}3\uparrow $$

For the systems, where the starting reagents were in the ratio PbI_2_:CH_3_NH_3_I = 1:2, after evaporation of the solvent, the formation of the additional phase (CH_3_NH_3_)_2_PbI_4_ has been observed (Fig. 4b). With the increasing of the temperature of heat treatment up to 180 °C, the decreasing of the intensity of this peak have been observed. At 180 °C, the resulting films were single-phase. The scheme of the reaction of formation of perovskite, where the starting reagents were taken in the ratios PbI_2_:CH_3_NH_3_I = 1:2, can be written as:

$$ \mathrm{Pb}{\mathrm{I}}_2+2\ \mathrm{C}{\mathrm{H}}_3\mathrm{N}{\mathrm{H}}_3\mathrm{I}\ \overset{20-120{}^{\circ}\mathrm{C}}{\to }{\left(\mathrm{C}{\mathrm{H}}_3\mathrm{N}{\mathrm{H}}_3\right)}_2\mathrm{Pb}{\mathrm{I}}_4\overset{>180{}^{\circ}\mathrm{C}}{\to } $$

3 $$ \overset{>180{}^{\circ}\mathrm{C}}{\to}\mathrm{C}{\mathrm{H}}_3\mathrm{N}{\mathrm{H}}_3\mathrm{Pb}{\mathrm{I}}_3+\kern0.5em \mathrm{C}{\mathrm{H}}_3\mathrm{I}\uparrow +\mathrm{N}{\mathrm{H}}_3\uparrow $$

When the starting reagents were in the ratio PbI_2_:CH_3_NH_3_I = 1:3, the intermediate phase (CH_3_NH_3_)_3_PbI_5_ was formed, as well as a phase (CH_3_NH_3_)_2_PbI_4_ (Fig. 4c). Intermediate phases (CH_3_NH_3_)_3_PbI_5_ and (CH_3_NH_3_)_2_PbI_4_ were described in [44, 45]. With the increasing of the temperature of heat treatment up to 170 °C, a single-phase perovskite structure is formed. The scheme of the reaction of formation of perovskite can be written as:

$$ \mathrm{Pb}{\mathrm{I}}_2+3\ \mathrm{C}{\mathrm{H}}_3\mathrm{N}{\mathrm{H}}_3\mathrm{I}\overset{20-120{}^{\circ}\mathrm{C}}{\to }{\left(\mathrm{C}{\mathrm{H}}_3\mathrm{N}{\mathrm{H}}_3\right)}_3\mathrm{Pb}{\mathrm{I}}_5\overset{>120{}^{\circ}\mathrm{C}}{\to } $$

$$ \overset{>120{}^{\circ}\mathrm{C}}{\to }{\left(\mathrm{C}{\mathrm{H}}_3\mathrm{N}{\mathrm{H}}_3\right)}_2\mathrm{Pb}{\mathrm{I}}_4+\mathrm{C}{\mathrm{H}}_3\mathrm{I}\uparrow +\mathrm{N}{\mathrm{H}}_3\uparrow \overset{>170{}^{\circ}\mathrm{C}}{\to } $$

4 $$ \overset{>170{}^{\circ}\mathrm{C}}{\to }\ \mathrm{C}{\mathrm{H}}_3\mathrm{N}{\mathrm{H}}_3\mathrm{Pb}{\mathrm{I}}_3+\kern0.5em \mathrm{C}{\mathrm{H}}_3\mathrm{I}\uparrow +\mathrm{N}{\mathrm{H}}_3\uparrow $$

With the increase of the temperature of the heat treatment up to 180 °C, thermal decomposition of perovskite in accordance with the chemical reaction (2), has been observed.

**Fig. 5:**
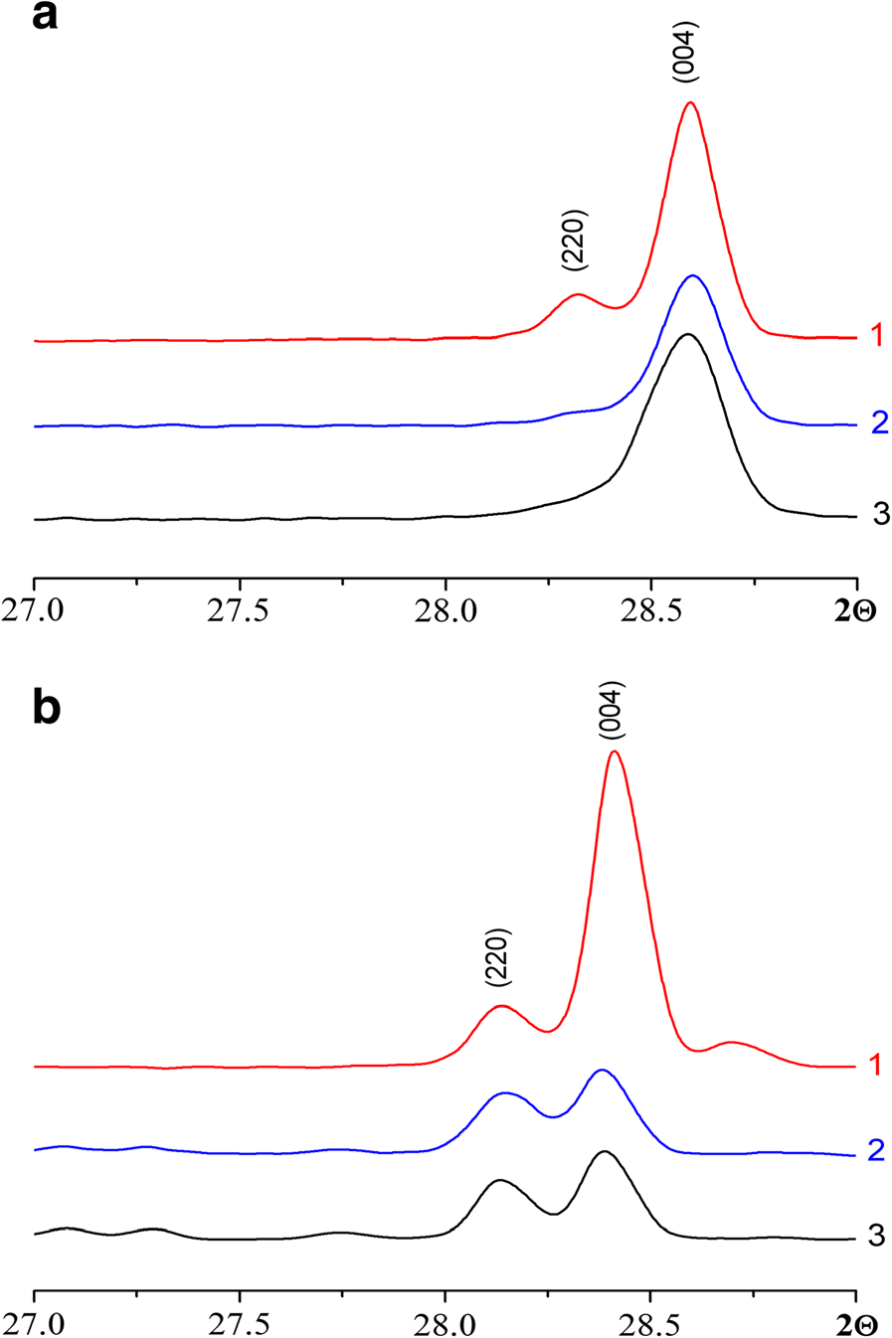
XRPD pattern of peaks 220 and 004 of CH_3_NH_3_PbI_3_ films deposited on the glass substrate (**a**) and on the FTO (**b**) at 85 °C at different ratios of the starting reagents PbI_2_ and CH_3_NH_3_I: 1:1 (1); 1:2 (2); 1:3 (3)

It is known that the perovskites of CH_3_NH_3_PbI_3_ can take three different phases: orthorhombic at temperatures below − 111 °С [46], tetragonal in the temperature range from − 110 to 51 °C, and cubic at temperatures above 51 °C [47]. In all of our systems (1:1, 1:2, 1:3), tetragonal symmetry (spatial group I4/mcm), which is confirmed by the splitting of peaks (220)/(004), has been observed (Fig. 5).

Figure 5 shows the XRPD diffraction in a narrow 2Θ range (27–29 °) for peaks (220) and (004), for films which were deposited on glass and FTO/glass substrates. The ratio of the intensities of these peaks depends on a number of factors: chemical composition, occupancy of positions in the structure, and anisotropy of the shape of particles. Previously, we have shown that independent on the ratio of the initial components (CH_3_NH_3_I:PbI_2_ = 1:1, 1:2, 1:3), the ratio between the content of lead and iodine in the films remains unchanged [20]. As has been shown by the calculations, the greatest contribution to the intensity gives the heaviest element—lead (in the ratio 1:2). But for a sample synthesized at a ratio of the starting reagents 1:1, the ratio of the intensities of the peaks is significantly greater than in case of system 1:2. Such a significant difference in the intensity of the peaks (220) and (004) could be explained only by the anisotropy of the particle shape, which is consistent with the data of electron microscopy (Fig. 3). Suitably, the shape of the particles for the sample PbI_2_:CH_3_NH_3_I = 1:1 deposited on the glass substrate is strongly anisotropic (see Fig. 3a). For samples synthesized at a ratio of the initial reagents 1:2 and 1:3, the ratios of the intensities of the peaks (220) and (004) practically the same, which is consistent with the small anisotropy of the particles or its absence (see Fig. 3 b, c respectively). Similar results are observed for samples, where films are deposited on the FTO/glass substrate.

For a more detailed study of the influence of initial reagents ratio PbI_2_:CH_3_NH_3_I and the temperature of crystallization of the film on the formation of perovskites structure, Raman spectroscopy was carried out.

**Fig. 6:**
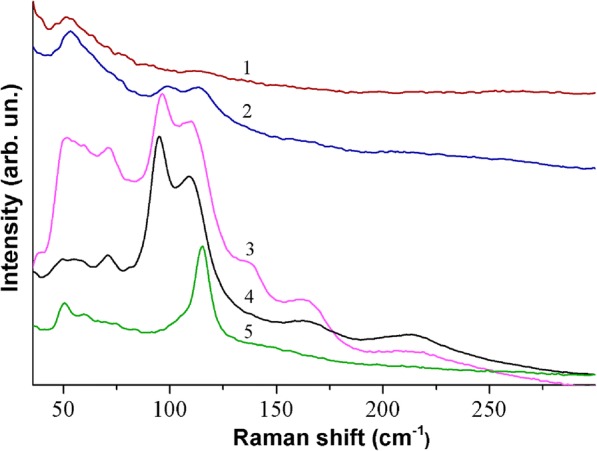
Raman spectra of CH_3_NH_3_PbI_3_ films formed of the 1:1 mixture of PbI_2_ and CH_3_NH_3_I in DMF: directly after deposition on a glass substrate at *T* = 90 °C (1); after irradiation by laser light for 200 s (2); after irradiation for 400 s (3). Raman spectra of films formed from the solution of pure PbI_2_ (4) and CH_3_NH_3_I (5) in DMF. All spectra were obtained with *λ*_exc_ = 532 nm at room temperature

Figure 6, curve 1 shows Raman spectrum of the CH_3_NH_3_PbI_3_ film formed from the solution of the PbI_2_ and CH_3_NH_3_I compounds in the ratio (1:1) in DMF and is registered at a sufficiently small power of exciting laser radiation (~ 5 × 10^2^ W/cm^2^). The spectra of CH_3_NH_3_PbI_3_ films, formed from the solution of PbI_2_ and CH_3_NH_3_I in DMF which are taken in the ratio 1:1, 1:2, and 1:3, are similar and are not shown for the last two films (spectra are shown in Additional file 1). This suggests that despite the different film morphology [32], their structural units are the crystalline lattice of tetragonal perovskite. As noted above, perovskite films are quite sensitive to external factors (moisture, intense X-ray, and laser radiation). When films were irradiated with exciting laser radiation for 200 s, the Raman spectrum changes significantly (Fig. 6, curve 2). A similar change in the spectra occurs when the power density of the exciting laser radiation increases by about five times. With this effect of laser radiation, the CH_3_NH_3_PbI_3_ film transforms into a metastable state, which is a transitional state from the perovskite to the PbI_2_. Indeed, intense laser radiation can lead to the destruction of chemical bonds in CH_3_NH_3_PbI_3_, and to the excitation of the electronic subsystem of individual structural units, which contributes to the formation of a metastable structure. In particular, such a state may be the result of the intercalation of the compound formed due to the partial destruction of the perovskite into PbI_2_ [44]. The change in the form of Raman spectra of films in such a metastable state is observed directly in the process of their measurements. In particular, after an additional irradiation of the film by laser radiation during 200 s, the Raman spectrum has significantly changed (Fig. 6, curve 2). In Fig. 6, for comparison, the spectra of films formed by the deposition of DMF solution with PbI_2_ (curve 4) and CH_3_NH_3_I (curve 5) compounds are also given. Further increase in the time of irradiation of the CH_3_NH_3_PbI_3_film by laser radiation with the same power leads to the complete destruction of CH_3_NH_3_PbI_3_. As a result, the spectrum is similar to curve 4, in Fig. 3, which corresponds to the vibrational spectra of the PbI_2_ compound. It should be noted that the destruction of the CH_3_NH_3_PbI_3_ perovskite during its interaction with moisture is much slower [48].

The results of X-ray diffraction analysis showed that when the ratio of the initial reagents PbI_2_:CH_3_NH_3_I = 1:2 and 1:3, the formation of the perovskite structure occurs through intermediates (CH_3_NH_3_)_3_PbI_5_ and (CH_3_NH_3_)_2_PbI_4_. In Raman spectra, it is difficult to detect these compounds, since the frequencies of the vibrational modes of CH_3_NH_3_PbI_3_, (CH_3_NH_3_)_3_PbI_5_ and (CH_3_NH_3_)_2_PbI_4_ in the low-frequency region of the spectrum are quite close [49].

**Fig. 7:**
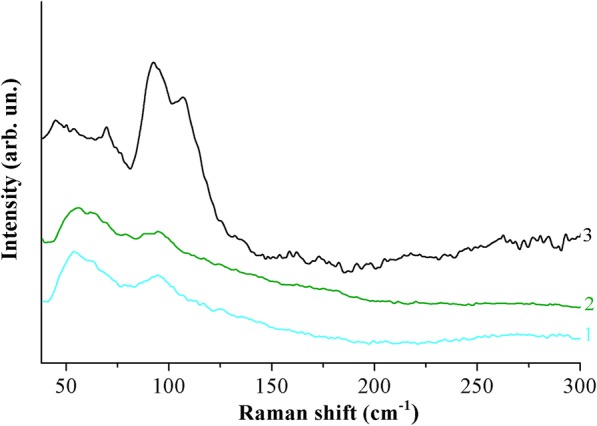
Raman spectra of the films formed of the solution of PbI_2_ and CH_3_NH_3_I compounds in DMF in the ratio (1:3) at temperatures of 100 (1), 150 (2), and 180 °C (3). All spectra were obtained with *λ*_exc_ = 532 nm at room temperature

We also carried out Raman studies of perovskite films formed from solutions of PbI_2_ and CH_3_NH_3_I compounds (1:3) in DMF, which were annealed in the temperature range from 100 to 180 °C (Fig. 7). The spectra of films that were treated at temperatures up to 180 °C are quite similar to the spectrum 1, which is shown in Fig. 6. However, the Raman spectrum of the film that was treated at *T* = 180 °C already corresponds to the spectrum of the metastable phase (curve 3 in Fig. 6). These results correlate with the data of X-ray diffraction analysis.

## Conclusions

Therefore, the possibility to control morphology, structural, and optical properties of CH_3_NH_3_PbI_3_ films by variation of the ratio of initial compounds, of PbI_2_ and CH_3_NH_3_I in DMF solvent, was found. X-ray diffraction analysis has shown that the formation of the perovskite structure with the ratio of the initial reagents PbI_2_: CH_3_NH_3_I = 1:1 occurs at 70–80 °C, and with the increase of the temperature of thermal treatment to 120 °C, the thermal destruction of the perovskite begins. When the ratio of the starting reagents PbI_2_: CH_3_NH_3_I = 1:2, the formation of the perovskite structure occurs through the intermediate compound (CH_3_NH_3_)_2_PbI_4_, and when the ratio is 1:3—(CH_3_NH_3_)_3_PbI_5_ and (CH_3_NH_3_)_2_PbI_4_. Independent on the ratio of the initial components (CH_3_NH_3_I:PbI_2_), the ratio between the content of lead and iodine in the films remains unchanged, that is why a significant difference in the film properties could be explained by the anisotropy of the particle shape, which is consistent with the data of electron microscopy, as well as with X-ray diffractometry (change in the ration of peaks (220) and (004) intensity). By using Raman spectroscopy, it was shown that films are sensitive to laser radiation, which leads to destruction, the final product of which is PbI_2_. When illuminated with laser radiation with low power density, they may be in a metastable state for some time, which is a transition from perovskite to PbI_2_.

## Additional file

Additional file 1: **Figure S1.** UV-vis absorption spectra of solutions: 1 – PbI_2_; 2 – PbI_2_ and CH_3_NH_3_I (1:1); 3 – PbI_2_ and CH_3_NH_3_I (1:2); 4 – PbI_2_ and CH_3_NH_3_I (1:3) in DMF. Figure S2 Raman spectra of the films formed of the solution of PbI_2_ and CH_3_NH_3_I in DMF in the ratio 1:1 (1); 1:2 (2); and 1:3 (3) at 90 °C. All spectra were recorded with λ_exc_ = 532 nm at room temperature. Figure S3 (a) Back-scattered electrons (BSE) images of heterogeneity on the surface of CH_3_NH_3_I films prepared at room temperature (no heating). (b) Energy-dispersive X-ray (EDX) spectra of the region selected within the heterogeneity asea (Selected Area 1) and outside of heterogeneity (Selected Area 2). (c) Cross-section of the film CH_3_NH_3_I on the surface of the glass in the area of heterogeneity. (DOCX 894 kb)

## Abbreviations

BSE: Backscattering electrons
CCD: Charge-coupled device
DMF: Dimethylformamide, C_3_H_7_NO
EDX: Energy-dispersive X-ray spectroscopy
FTIR: Fourier-transform infrared spectroscopy
FTO: Fluorine-doped tin oxide
MAI: Methylammonium iodide
MAPbI_3_: Methylammonium lead iodide perovskites, CH_3_NH_3_PbI_3_
MAPbI_3-x_Cl_x_: Methylammonium lead iodide chloride perovskites, CH_3_NH_3_PbI_2.98_Cl_0.02_
OIP: Organic-inorganic perovskites
PCE: Power conversion efficiencies
XRPD: X-ray powder diffractometry
