# Supplementary material for: Effect of non-stoichiometry of initial reagents on morphological and structural properties of perovskites CH3NH3PbI3
Source: Nanoscale Res Lett. 2019 Jan 5;14:4. doi: 10.1186/s11671-018-2841-6 (PMC6320708; doi:10.1186/s11671-018-2841-6)
Supplement: Supplementary file 1 — Figure S1. UV-vis absorption spectra of solutions: 1 – PbI2; 2 – PbI2 and CH3NH3I (1:1); 3 – PbI2 and CH3NH3I (1:2); 4 – PbI2 and CH3NH3I (1:3) in DMF. Figure S2 Raman spectra of the films formed of the solution of PbI2 and CH3NH3I in DMF in the ratio 1:1 (1); 1:2 (2); and 1:3 (3) at 90 °C. All spectra were recorded with λexc = 532 nm at room temperature. Figure S3 (a) Back-scattered electrons (BSE) images of heterogeneity on the surface of CH3NH3I films prepared at room temperature (no heating). (b) Energy-dispersive X-ray (EDX) spectra of the region selected within the heterogeneity asea (Selected Area 1) and outside of heterogeneity (Selected Area 2). (c) Cross-section of the film CH3NH3I on the surface of the glass in the area of heterogeneity. (DOCX 894 kb) [file 11671_2018_2841_MOESM1_ESM.docx]

**Supplementary information**


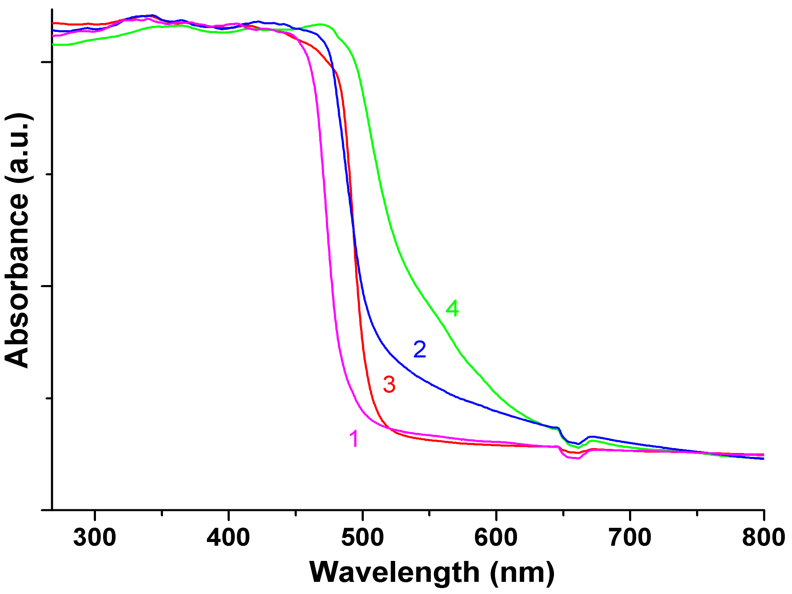


**Fig. S1.** UV-vis absorption spectra of solutions: 1 – PbI_2_; 2 – PbI_2_ and CH_3_NH_3_I (1:1); 3 – PbI_2_ and CH_3_NH_3_I (1:2); 4 – PbI_2_ and CH_3_NH_3_I (1:3) in DMF.


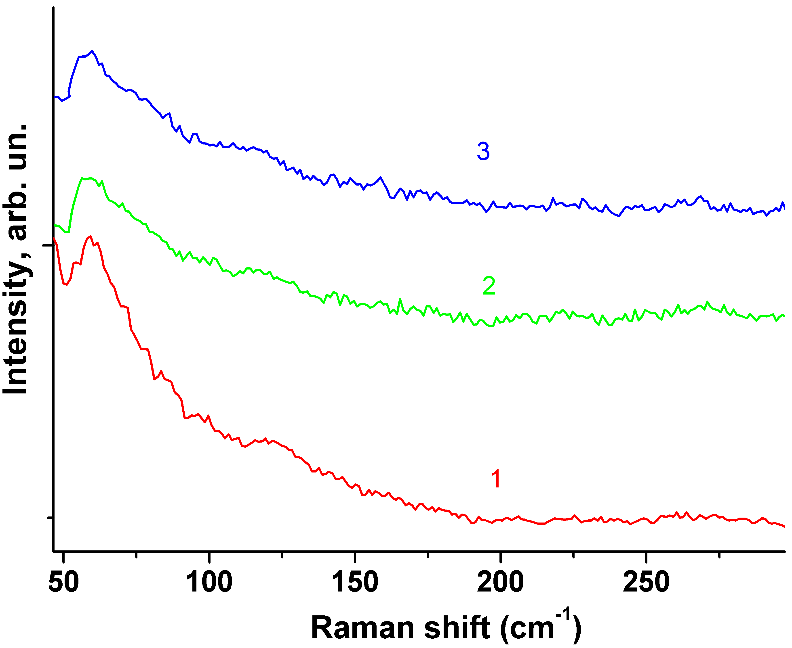


**Fig. S2.** Raman spectra of the films formed of the solution of PbI_2_ and CH_3_NH_3_I in DMF in the ratio 1:1 (1); 1:2 (2); and 1:3 (3) at 90 °C. All spectra were recorded with λ_exc_=532 nm at room temperature.


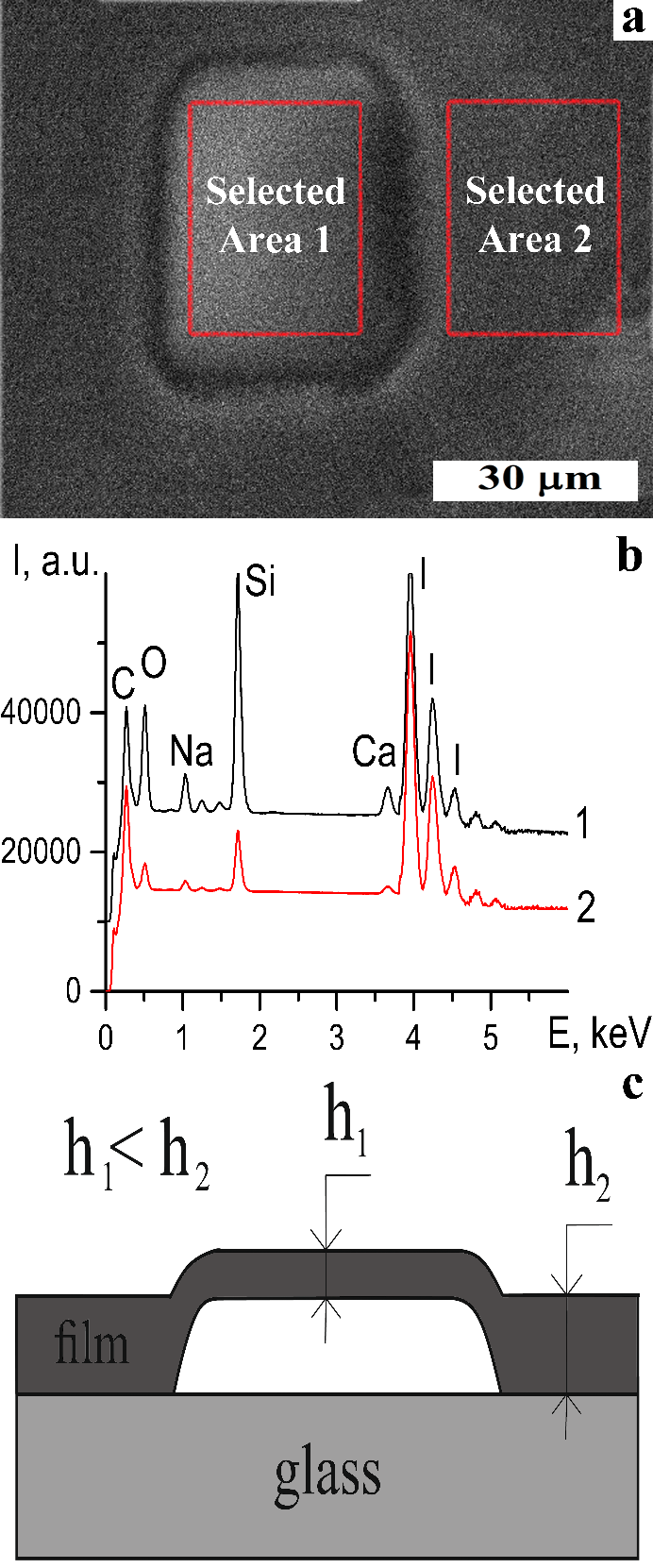


**Fig. S3.** (a) Back-scattered electrons (BSE) images of heterogeneity on the surface of CH_3_NH_3_I films prepared at room temperature (no heating).

(b) Energy-dispersive X-ray (EDX) spectra of the region selected within the heterogeneity asea (Selected Area 1) and outside of heterogeneity (Selected Area 2).

(c) Cross-section of the film CH_3_NH_3_I on the surface of the glass in the area of heterogeneity.

Fig. 3a shows CH_3_NH_3_I film deposited to the glass surface at room temperature. The film contains a small number of heterogeneities. Fig. 3b shows the EDX spectra of the surface of CH_3_NH_3_I films in the area of heterogeneity (Selected Area 1) and outside of heterogeneity (Selected Area 2). EDX (Fig. 3b) showed that the intensity of the peaks of elements contained in the glass (O, Na, Si, Ca) in the region of heterogeneity is significantly higher (curve 1) than outside of heterogeneity of the film (curve 2). This fact can be explained by the less thickness of the film in the region of heterogeneity (Fig. 3c), which is formed during the rapid evaporation of the solvent. Fig. 3c shows proposed cross-section of the film CH_3_NH_3_I on the glass surface in the area of heterogeneity.
